# Interactive effects of low-density polyethylene microplastics and benzo[a]pyrene on the growth of the freshwater microalgae *Raphidocelis subcapitata*

**DOI:** 10.1007/s10646-026-03143-3

**Published:** 2026-09-01

**Authors:** Yuuri Gabriel de Souza Santos, Rodrigo Brasil Choueri, Caio Rodrigues Nobre, Fábio Ruiz Simões, Paloma Kachel Gusso-Choueri

**Affiliations:** 1 Laboratório de Ecotoxicologia, Universidade Santa Cecília (Unisanta), Rua Oswaldo Cruz, 266, São Paulo, 11045-907 Santos Brazil; 2 Departamento de Ciências do Mar, Instituto do Mar, Universidade Federal de São Paulo, Campus Santos (Unifesp), Rua Carvalho de Mendonça, 144, São Paulo, 11070-102 Santos Brazil

**Keywords:** Polyethylene, PAHs, Toxicity, Microplastics, *Raphidocelis subcapitata*

## Abstract

Although microplastics (MPs) and polycyclic aromatic hydrocarbons (PAHs) are widespread in aquatic environments, their combined effects in freshwater systems remain poorly understood. This gap is relevant because green algae, frequently exposed to MPs, play an important role in PAHs bioremediation. This study evaluated how MPs influence the toxicity of benzo[a]pyrene (BaP) in the green microalga *Raphidocelis subcapitata*. A full-factorial design was used to expose algal cells to three concentrations of low-density polyethylene MPs (5, 50, and 500 mg L⁻¹), tested alone or in combination with the BaP median effective concentration (EC50-72 h) determined for this species. Experiments lasted 72 ± 2 h under controlled temperature, agitation, and light. The isolated and combined effects of MPs and BaP were analyzed using Generalized Linear Models. The EC50 of BaP was 21 µg L⁻¹ (20–25 µg L⁻¹) and the range provided refers to 95% confidence interval (CI_95%_). When tested separately, MPs at 5 and 50 mg L⁻¹ reduced algal density, likely due to additives associated with the particles. No inhibitory effect was observed at 500 mg L⁻¹, possibly because the particles served as a surface for biofilm formation, supporting growth. In combined exposures, the presence of MPs at 50 and 500 mg L⁻¹ reduced BaP toxicity, consistent with increased sorption of hydrophobic compounds to plastic surfaces and reduced BaP availability in the medium. In contrast, treatments combining BaP with 5 mg L⁻¹ MPs showed greater toxicity than BaP alone, suggesting that this MP concentration was insufficient to remove a meaningful fraction of BaP from the water, allowing interaction with cellular membranes and growth inhibition. These results indicate that the influence of MPs on BaP toxicity is concentration-dependent and nonlinear, suggesting that MPs can either enhance or mitigate BaP toxicity depending on contamination levels. Such interactions may alter the environmental bioavailability of PAHs in freshwater ecosystems, highlighting the importance of considering MP concentrations in ecological risk assessments.

**Supplementary Information:**

The online version contains supplementary material available at https://doi.org/10.1007/s10646-026-03143-3.

## Introduction

PAHs are compounds consisting of two or more aromatic rings (Zena et al. 2015), mainly generated by the combustion of fossil fuels, plant biomass, and petroleum derivatives (Tomar and Jajoo 2021). They are considered persistent environmental contaminants and exhibit a high affinity for particulate materials, such as MPs (Bartonitz et al. 2020; Godoi et al. 2004). PAHs can induce toxicity and oxidative stress in green algae by stimulating the excessive production of reactive oxygen species (ROS) through mechanisms associated with cellular electron transport (Wang et al. 2013). Moreover, these compounds are highly toxic and have been associated with several adverse effects in aquatic organisms, including narcosis, cardiac dysfunction, mutagenicity, and carcinogenicity in fish (Martins et al. 2020). Among PAHs of greater ecological relevance, benzo[a]pyrene (BaP) stands out as one of the most potent genotoxic, bioaccumulative, and teratogenic agents in aquatic environments (Li and Chen 2002; Garcia de Llasera et al.,^ 2016^).

Although PAHs contamination in marine environments has been extensively studied, investigations into their impact on freshwater systems remain scarce (Szopińska et al. 2019; Sandil et al. 2025). PAHs contamination in ecosystems rarely occurs in isolation, being frequently associated with other contaminants (Wang et al. 2013). In this context, MPs are also abundant in aquatic environments. When considering ecotoxicological studies that address the combined effects of these contaminants, findings remain even more limited (Sun et al. 2021; Davey et al. 2023). These contaminants may enter freshwater ecosystems through surface runoff, contamination of groundwater and bottom sediments, direct spills, improper waste disposal, and atmospheric deposition. Furthermore, their entry into the aquatic food web may occur through bioaccumulation in algae, thereby making these contaminants available to organisms at higher trophic levels (Mahaney 1994; Alimba and Fagio, 2019; Tomar and Jajoo 2021; Shi et al. 2022).

Since both contaminants are frequently introduced into marine environments through rivers (Lagarde et al. 2016; Wu et al. 2019; Chen et al. 2019), this highlights the importance of expanding research on species inhabiting freshwater ecosystems using environmentally realistic concentrations. Algae have been widely used in ecotoxicological studies due to their ease of cultivation, low nutritional requirements, high cell division rate, and elevated sensitivity to contaminants (Zhao et al. 2018). Moreover, they play an essential role as primary producers in aquatic ecosystems (Machado and Soares 2022). Changes in algal populations may compromise the structure and functioning of food webs through the transfer of energy and matter across trophic levels (Abinandan et al. 2023). Green algae, such as *Raphidocelis subcapitata*, are of great ecological importance and play a key role in the remediation of sites contaminated by PAHs (Othman et al. 2023; Garcia and García de Llasera,^ 2023^). However, the impact of microplastics in these contaminated environments remains unknown, raising concerns about the effectiveness of bioremediation in scenarios where both contaminants co-occur (Machado and Soares 2024). In this context, it is necessary to understand their responses to binary mixture of contaminants, with the aim of assessing both their effectiveness in bioremediation processes and their ability to recover from the toxicity effects (Wang et al. 2013).

Accordingly, the general objective of this study was to evaluate the effect of combined exposure to low-density polyethylene microplastics and BaP in *Raphidocelis subcapitata*. For this purpose, the algae were exposed to the BaP EC50 concentration, defined as the concentration that inhibits algal biomass by 50% under the experimental conditions, in combination with three different microplastic concentrations. The exposure started with an environmentally relevant concentration (5 mg L⁻¹), previously reported in freshwater environments (Antic et al., 2011; Van Wezel et al. 2016; Lasee et al. 2017) and also included higher concentrations to simulate a more extreme scenario to MP exposure (50 and 500 mg L⁻¹). The tested hypothesis was that microplastics could alter the toxicity of BaP to this microalgal species by changing its bioavailability in the exposure medium, potentially increasing or reducing its toxic effects depending on microplastic concentration.

## Materials and methods

### Culture conditions

The cultivation of the microalga species *Raphidocelis subcapitata* followed the guidelines established by NBR 12,648 (ABNT, 2018). The strain used in the present study was obtained from cultures previously maintained at the Ecotoxicology Laboratory of Universidade Santa Cecília.

The cultures were prepared under sterile conditions by inoculating 1 mL of algal suspension containing at least 10⁶ cells into 250 mL Erlenmeyer flasks containing 100 mL of L.C. Oligo culture medium (reagents and concentrations are described in the Supplementary Material S1). Both the culture medium and glassware were previously sterilized by autoclaving.

An aliquot of 0.2 mL of the culture was transferred to a Neubauer chamber for the initial cell count under an optical microscope. The cultures were then maintained in a growth chamber under continuous illumination (4000 lx), at 24 ± 1 °C, with daily agitation for seven days. After the incubation period, a new aliquot was transferred to a Neubauer chamber for the final algal cell count under an optical microscope. Fresh cultures were established weekly following the same procedure described above.

### Preparation of chemical solutions

Stock solutions of BaP (Sigma-Aldrich^®^, ≤ 96% HPLC purity) were prepared using analytical-grade acetone (0.015%) suitable for pesticide residue analysis as the solvent. All solutions were prepared at the Ecotoxicology Laboratory of Universidade Santa Cecília using calibrated equipment, including pipettes and glassware, in accordance with the standards of the Brazilian Calibration Network (RBC). To minimize potential degradation losses, all solutions were prepared on the same day the experiments were conducted.

The methodology followed the guidelines established by NBR 12,648 (ABNT, 2018) for chronic toxicity tests with green algae. In parallel with the experiments, sensitivity tests were performed using zinc sulfate heptahydrate (ZnSO₄·7 H₂O) as the reference substance. The sensitivity assay conducted with the batch of organisms used in the present study resulted in a 72 h EC₅₀ of 0.005 mg L⁻¹, which was within the limits established by the laboratory control chart (acceptable sensitivity range: 0.003–0.007 mg L⁻¹ ZnSO₄).

#### Determination of BaP EC_50_

Prior to the definitive assay, preliminary tests were conducted to determine the median effective concentration (EC₅₀) for *Raphidocelis subcapitata*, defined as the concentration that produces 50% of the maximum effect under defined experimental conditions. A pre-culture of *R. subcapitata* was established seven days before the experiments following the procedure described in Sect.  2.1. At the end of this period, an aliquot was collected for cell counting using a Neubauer chamber under a light microscope to determine the final culture density and calculate the inoculum volume required for the preliminary tests.

The BaP concentrations tested (3, 12, 21, 30, and 39 µg L⁻¹) were selected based on EC₅₀ values reported in the literature for other aquatic organisms, including fish, sea urchins, and bacteria (Beiras et al., 2016; Bour et al. 2020; Batel et al. 2020; Su et al., 2021; de Mello e Souza et al., 2023), due to the lack of toxicity data available for *R. subcapitata*. The tested concentrations included environmentally relevant levels previously detected in rivers and lakes (2.36 and 3.32 µg L⁻¹) (Maskaoui et al. 2002; Sun et al. 2009), as well as higher concentrations potentially capable of inducing more pronounced toxic effects in microorganisms. For treatment preparation, sterilized L.C. Oligo culture medium was used (Table S1). Each treatment was prepared in triplicate using 250 mL Erlenmeyer flasks containing 100 mL of medium. BaP and acetone were added to the medium and solutions were gently homogenized. Subsequently, the required volume of inoculum was added to achieve an initial concentration of 10⁶ cells mL⁻¹, keeping the final volume at 100 mL per flask. Controls included: (i) culture medium control (organisms exposed only to standard medium), and (ii) solvent control (organisms exposed to medium with acetone).

The pH of controls was measured at the beginning and end of the experiment to monitor medium stability. The initial pH for the controls was 6.67. Treatments were randomly distributed in the culture chamber with daily alternation of flask positions to minimize light and temperature variations. The assay lasted 72 ± 2 h at 24 ± 1 °C, under continuous illumination of 4,500 lx with daily agitation. At the end of exposure, aliquots of each replicate were fixed with Lugol’s 5% solution. The initial biomass of each replicate was subtracted from the final biomass after exposure to calculate algal density. The toxicity factor (TF) was calculated from the mean biomass at each concentration and the inhibition percentage relative to the control, according to the equation:

$$\:Cl=\frac{(Mc-Ma)}{Mc}\times\:100$$

where:

Cl = inhibition percentage of algal cell multiplication;

Ma = mean number of cells in test solutions;

Mc = mean number of cells in the control.

#### Preparation of BaP and MPs solution

In a sterile environment, the respective contaminant concentrations were added to each treatment. For the BaP treatment, 100 mL of sterilized L.C. Oligo medium (Table S1) was transferred into 250 mL Erlenmeyer flasks, followed by the addition of the BaP EC₅₀ concentration to each flask. For the treatments containing only MPs, 100 mL of sterilized L.C. Oligo medium was added to 250 mL Erlenmeyer flasks, and the respective MP concentrations (5, 50, and 500 mg L⁻¹) were added to each container. For the combined BaP and MP treatments, the same procedure was followed, with the addition of both the respective MP concentration and the BaP EC₅₀ concentration to each flask.

### Experimental design

Organisms were exposed to the BaP EC₅₀ concentration in combination with three microplastic (MP) concentrations (5, 50, and 500 mg L⁻¹; approximately 1 × 10³, 1 × 10⁴, and 1 × 10⁵ particles L⁻¹, respectively). The tested MP concentrations included an environmentally relevant concentration (5 mg L⁻¹) previously reported in freshwater environments (Antic et al., 2011; Van Wezel et al. 2016; Lasee et al. 2017). The experiments were conducted using a full-factorial design, in which all possible combinations of both factors were simultaneously evaluated.

Three replicates were prepared for each treatment and control condition: (i) culture medium control; (ii) solvent control (acetone); (iii) BaP only; (iv) 5 mg L⁻¹ MPs; (v) 50 mg L⁻¹ MPs; (vi) 500 mg L⁻¹ MPs; (vii) BaP + 5 mg L⁻¹ MPs; (viii) BaP + 50 mg L⁻¹ MPs; and (ix) BaP + 500 mg L⁻¹ MPs.

After preparation, the algal inoculum was added to obtain an initial density of 10⁶ cells mL⁻¹, with a final volume of 100 mL per flask. The solutions were gently homogenized and randomly distributed within the culture chamber, with daily alternation of flask positions to minimize spatial effects. The assay lasted 72 ± 2 h at 24 ± 1 °C under continuous illumination (4,500 lx) with daily manual agitation.

The pH of the culture medium controls was measured at the beginning and end of the assay, and variations remained below 1.5 units. At the end of the experiment, samples were preserved with 5% Lugol’s solution and analyzed under a light microscope for algal biomass determination by cell counting using a Neubauer chamber. The percentage of growth inhibition was subsequently calculated as previously described.

### Microplastic characterization

Microplastics particles were characterized by Fourier Transform Infrared Spectroscopy (FT-IR) using a Spectrum Two spectrophotometer (Perkin Elmer^®^) equipped with an ATR accessory (Universal Attenuated Total Reflection). Analyses were performed with Spectrum 10 software under the following parameters: spectral range 4,000–550 cm⁻¹, data interval 1 cm⁻¹, resolution 4 cm⁻¹, and 16 accumulative scans.

**Fig. 1:**
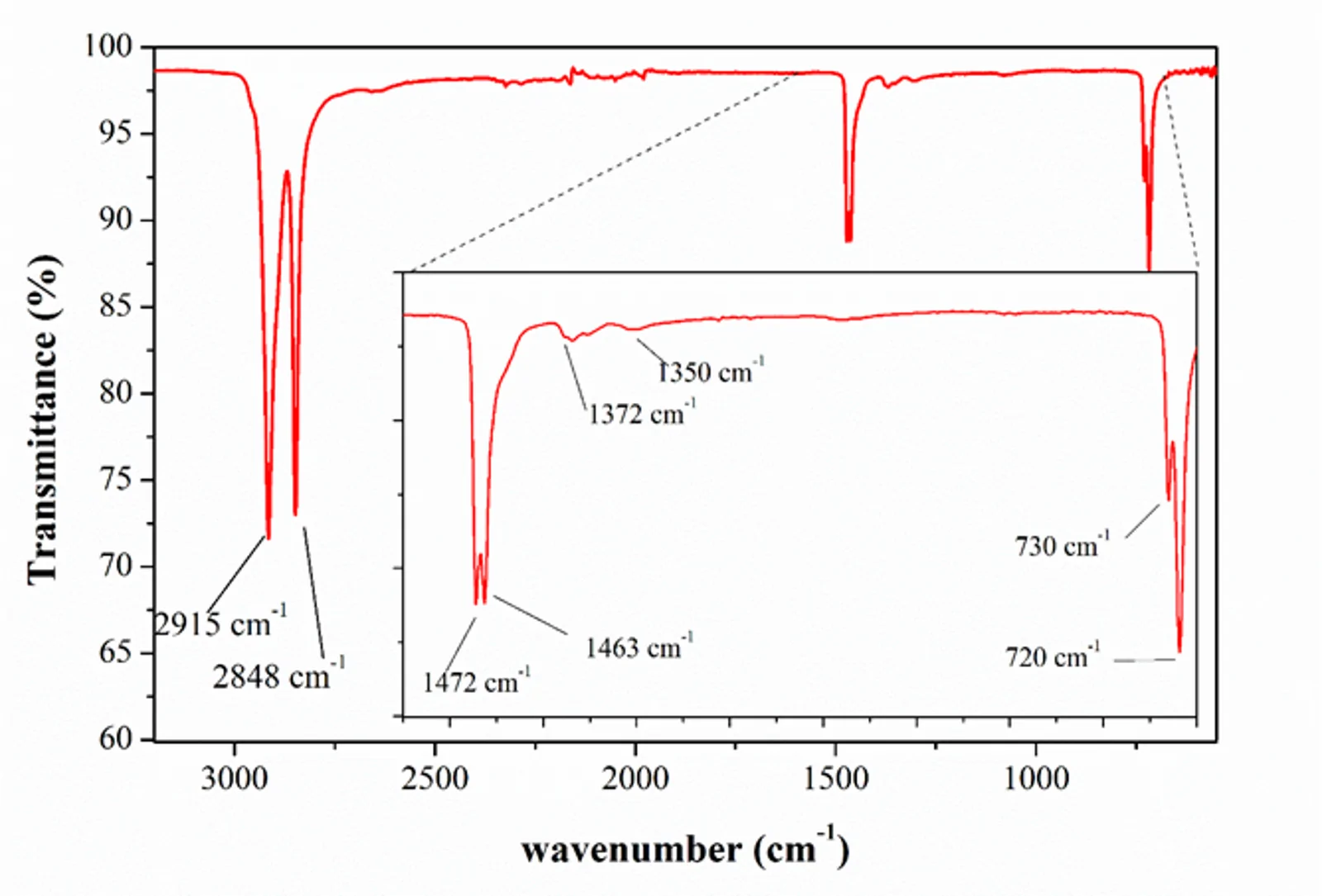
Results obtained from the FT-IR spectrum (ATR) about the characterization of microplastic

Figure 1 presents the FT-IR spectrum of polyethylene microspheres used in the current research. The bands located at 2915 cm⁻¹ and 2848 cm⁻¹ correspond to the asymmetric and symmetric stretching of CH₂ bonds, respectively. The bands at 1472 cm⁻¹ and 1463 cm⁻¹ are associated with CH₂ bending vibrations, while those at 730 cm⁻¹ and 720 cm⁻¹ correspond to CH₂ rocking motions. It is common for crystalline polymers to exhibit split peaks in the FT-IR spectrum, as observed in the regions of 1472/1463 cm⁻¹ and 730/720 cm⁻¹, which are typical features of high-density polyethylene (HDPE).

Additionally, the bands at 1372 cm⁻¹ and 1350 cm⁻¹ were assigned to the symmetric bending and wagging modes of methyl groups (CH₃), respectively, suggesting the presence of low-density polyethylene (LDPE) (Gulmine et al. 2002). However, linear low-density polyethylene (LLDPE) may also display small peaks related to methyl groups at 1378 cm⁻¹, due to the presence of short side chains. Furthermore, the split peaks associated with CH₂ stretching and bending modes are also characteristic of this polymer type.

LDPE is synthesized with short side chains, predominantly ethyl groups, which promote parallel chain orientation and the formation of crystalline regions, resulting in the observed split peaks. Therefore, it is confirmed that the analyzed samples are composed of linear low-density polyethylene (LLDPE), as reported by the manufacturer (Smith 2021).

### Statistical analysis

The effective concentration of BaP that produced 50% inhibition of algal growth in *R. subcapitata* (median effective concentration, EC₅₀) was calculated using nonlinear regression. The regression model that best fit the data was selected based on the coefficient of determination (R²), the residual sum of squares, and the 95% confidence intervals, using GraphPad Prism software (version 5). Data normality was assessed using the Shapiro–Wilk test, while homoscedasticity was evaluated using Levene’s test. Differences among treatments exposed to different (BaP) concentrations and control group were analyzed by one-way analysis of variance (ANOVA), followed by Tukey’s post hoc test when significant differences were detected. For statistical comparison between the two control groups (culture medium control and solvent control), Student’s t-test was applied. All analyses considered a significance level of *p* < 0.05.

Statistical differences were analyzed using Generalized Linear Models (GLMs), considering each contaminant (BaP and MPs) as an independent variable and cell inhibition as the response variable, thus evaluating the isolated effect of each contaminant as well as potential interactions between them. Statistical interaction is defined as the combined effects of the factors (independent variables) on a variable of interest (dependent variable). If an interactive effect is observed, it means that the impact of one factor depends on the other, and vice versa (Stevens 1999, 2007). Data are expressed as mean ± SEM (standard error of the mean), and p-values < 0.05 (α) were considered statistically significant. Statistical analyses and graphical outputs were performed using GraphPad Prism^®^ 5.0 and Primer 6^®^.

## Results and discussion

### Determination of BaP EC₅₀

**Fig. 2:**
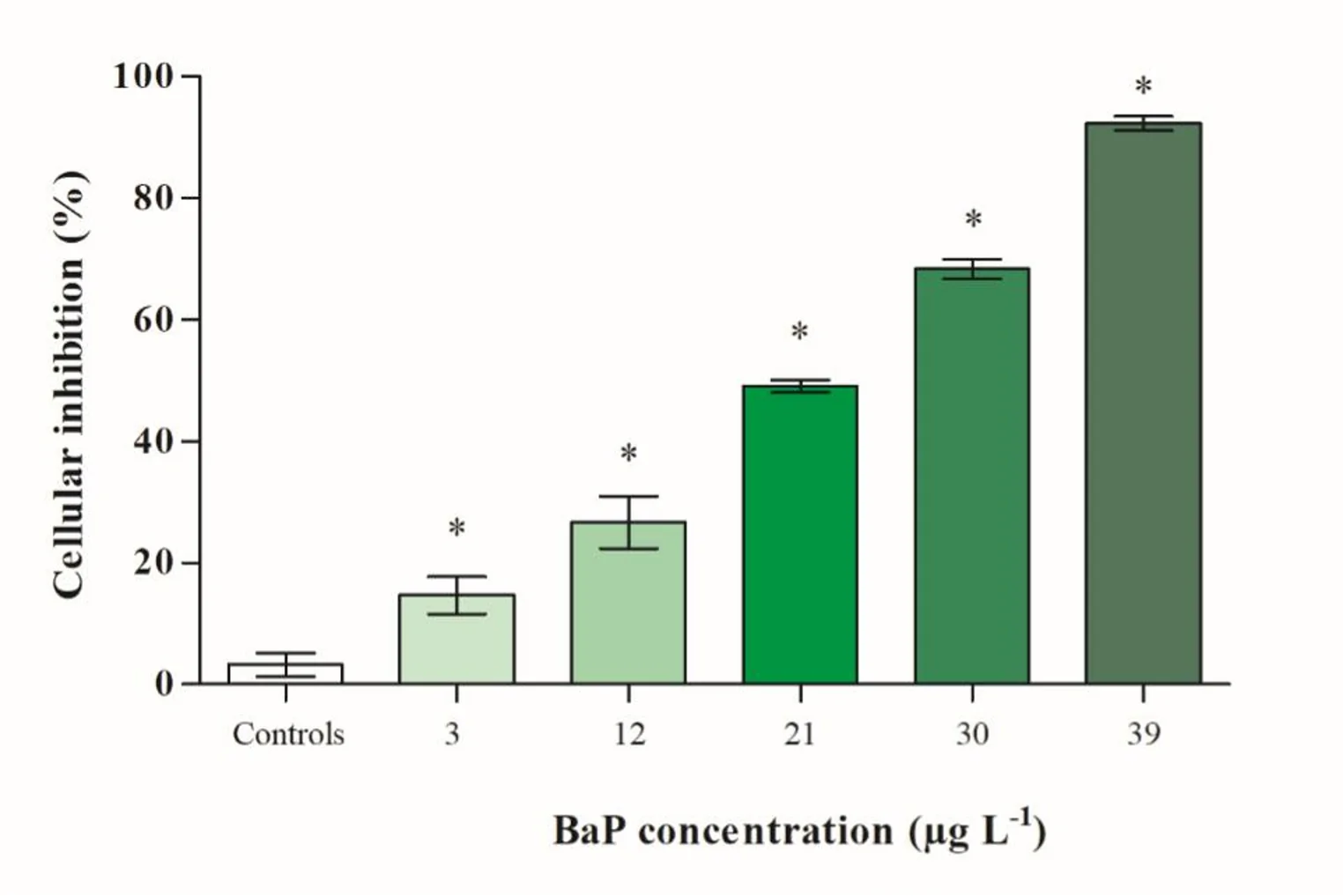
Effects of different benzo(a)pyrene (BaP) concentrations (µg L⁻¹) on the percentage of cellular inhibition in *Raphidocelis subcapitata*. Treatments included the control and BaP concentrations of 3, 12, 21, 30, and 39 µg L⁻¹. Data are presented as mean ± standard deviation. Asterisks (*) indicate statistically significant differences compared with the control treatment (*p* < 0.05)

The toxicity tests performed with different BaP concentrations demonstrated a concentration-dependent increase in cellular inhibition in *Raphidocelis subcapitata* (Fig. 2). Compared with the control group, all tested concentrations (3–39 µg L⁻¹) caused significant inhibition of algal growth (one-way ANOVA, *p* < 0.05). The lowest inhibition values were observed in the control treatment, whereas the highest BaP concentration tested (39 µg L⁻¹) resulted in inhibition values close to 90% (Fig. 2).

Based on the concentration-response curve obtained by nonlinear regression analysis, the estimated median effective concentration (EC₅₀) for BaP was approximately 21 µg L⁻¹, with a 95% confidence interval ranging from 20 to 25 µg L⁻¹. Statistical analyses also indicated that the lowest tested concentration (3 µg L⁻¹) already caused significant effects compared with the control treatment, indicating a LOEC (Lowest Observed Effect Concentration)) of 3 µg L⁻¹ for BaP exposure under the tested conditions (Fig. 2).

Although BaP has been detected in freshwater environments for several decades (Veldre et al. 1982), few studies have investigated its effects on organisms at the base of the food chain. It has been suggested that high concentrations of PAHs may also impair chlorophyll production in photosynthetic organisms, thereby reducing photosynthesis and consequently inhibiting growth once the cells become saturated with PAHs (Tomar and Jajoo 2021). Also, upon contact with BaP, this lipophilic compound is initially retained on the outer surface of the algae cell and subsequently internalized for metabolism (de Llasera et al., 2016)).

Previous studies indicate that *Raphidocelis subcapitata* is sensitive to BaP, with complete growth inhibition observed after 72 h of exposure to 160 µg L⁻¹ under white light conditions (Schoeny et al. 1988). In addition, an EC₅₀ of 1.6 µg L⁻¹ of BaP has been reported, but in its passive dosing exposure (with a constant substance release into the aqueous medium by equilibrium partitioning) (Kreutzer et al. 2022). The values obtained in the present study are consistent with EC₅₀ data reported for other freshwater microalgal species: *Chlorella fusca var. vacuolata* shows a higher sensitivity to BaP (EC₅₀ = 0.063 µg L⁻¹) (Grote et al. 2005) while *Chlorobion braunii* (formerly *Ankistrodesmus braunii*) shows a low sensitivity (EC₅₀ = 1300 µg L⁻¹) (Schoeny et al. 1988). Furthermore, *R. subcapitata* exhibits metabolic mechanisms capable of converting BaP into more soluble and less toxic compounds through the action of enzymes such as deoxygenases, which facilitates its excretion into the external medium (Othman et al. 2023). These findings suggest that although sensitive, *R. subcapitata* may exhibit some degree of tolerance to BaP, depending on environmental conditions such as light intensity (Warshawsky et al.,^ 1995^).

BaP is widely distributed in aquatic environmental matrices (de Llasera, 2016) and, like other PAHs, poses risks to organisms, being directly associated with phytoplankton toxicity in freshwater environments worldwide (Tomar and Jajoo 2021). Several studies have reported the presence of BaP in freshwater environments, with concentrations in the water column generally ranging from 0.0071 to 2.361 µg L⁻¹ (Sun et al. 2009; Liu et al. 2010; Zhang et al. 2012). Higher concentrations are frequently observed in freshwater surface sediments, ranging from 157.8 to 1750.04 µg kg⁻¹ dry weight (Liu et al. 2010; Patrolecco et al. 2010). Therefore, the EC₅₀ value estimated for *Raphidocelis subcapitata* in the present study (21 µg L⁻¹) is within the range of concentrations previously reported for contaminated freshwater environments.

### Effects of combined exposure to BaP and MP

After determining the EC_50_ of BaP, the subsequent assays with BaP-contaminated microplastics were conducted to test the interaction between the PAHs and MPs. First, the results of the two control groups were evaluated: (i) culture medium only, and (ii) culture medium with the solvent acetone. These two treatments showed no statistically significant differences; therefore, both treatments were combined and treated as a unique “control”. The following comparisons were made considering the difference relative to control.

**Fig. 3:**
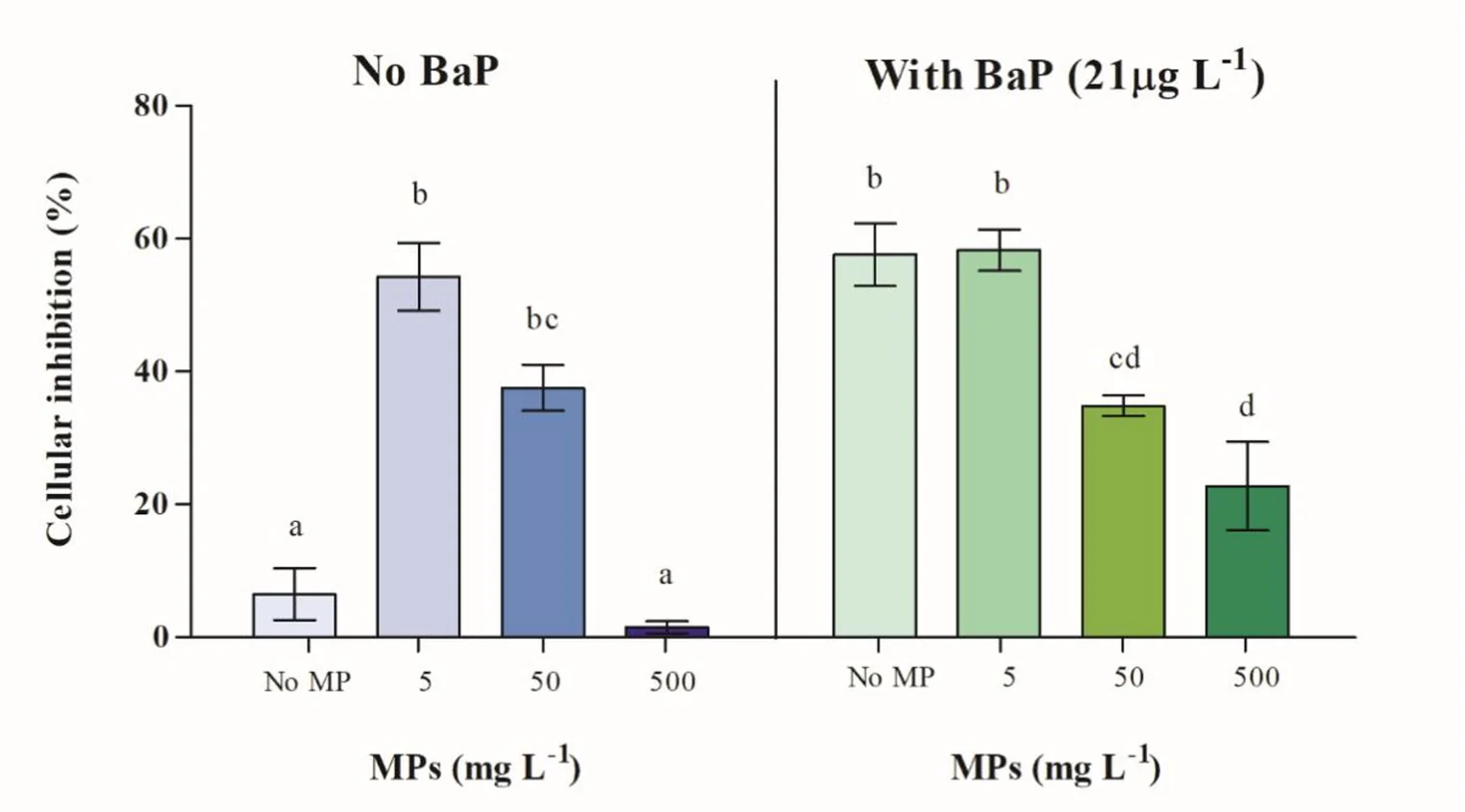
Cellular inhibition (%) in *Raphidocelis subcapitata* in response to the different treatments, including: solvent control, 5 mg L⁻¹ MP, 50 mg L⁻¹ MP, 500 mg L⁻¹ MP, BaP 21 µg L⁻¹, 5 mg L⁻¹ MP + BaP, 50 mg L⁻¹ MP + BaP, and 500 mg L⁻¹ MP + BaP. Data are presented as mean ± standard deviation. Different letters indicate statistically significant differences among treatments (*p* < 0.05)

As expected, when tested alone, the EC50 of BaP used (21 µg L⁻¹) caused greater algal inhibition compared to the solvent control, indicating that BaP at 21 µg L⁻¹ alone and the solvent control differ significantly (Fig. 3).

PAHs may be directly associated with toxicological responses in freshwater phytoplankton worldwide (Tomar and Jajoo 2021). The toxicity of BaP alone observed in the present study may be related to the interaction of BaP with microalgal membrane lipids, since PAHs are lipophilic compounds with high affinity for cell membranes and, consequently, can exert cytotoxic effects on the organism (Carvalho et al. 2011; Li et al. 2021). Some authors have reported an increase in lipid concentration in the cells of *R. subcapitata* and other green algal species in response to contaminant exposure (Carvalho et al. 2011; Tomar and Jajoo 2021). Although lipid quantification was not performed in the present study, the apparent formation of lipid clumps on the external surface of cells exposed to BaP was occasionally observed during routine microscopic examination. Such behavior can be considered a strategy to mitigate the cytotoxic damage caused by exposure to PAHs (Carvalho et al. 2011; Tomar and Jajoo 2021).

The results of this study, analyzed using GLMs, showed homogeneous variances and normally distributed residuals. As shown in Fig. 3, treatments containing only virgin MPs (without BaP contamination) (5, 50, and 500 mg L⁻¹) elicited two distinct responses. First, algal biomass was greater in the treatment containing the highest MP concentration (500 mg L⁻¹), exceeding the control group by 3.7% and the 5 and 50 mg L⁻¹ treatments by 57.9% and 42.4%, respectively. This absence of toxicity may be related to biofilm formation, as microplastics can provide a suitable surface for the development of microorganisms, including microalgae (Canniff and Hoang 2018; Chen et al. 2019). This interpretation is supported by Mizukami-Murata et al. (2021), who reported that millimeter-sized MPs did not inhibit the biomass of *R. subcapitata*. However, different responses may occur under exposure to nanoplastics (Mizukami-Murata et al. 2021; Bhatt & Chauhan, 2023), since smaller particles may contribute to cell wall degradation through adsorption onto the algal surface (Podbielska and Szpyrka 2023).Nevertheless, the strong affinity of algae to MP surfaces may facilitate the availability and transfer of these particles to other consumer organisms (Bhatt & Chauhan, 2023).

Regarding the results observed from the exposure of microalgae to virgin MPs, at concentrations of 5 mg L⁻¹ and 50 mg L⁻¹, an increase in algal cell inhibition was observed compared to the control treatment (Fig. 3). This toxicity may be associated with the presence of additives in the polymers (Cousin et al. 2020). Additives incorporated during polymer production ensure specific plastic properties, such as color, transparency, flexibility, and resistance to thermal, mechanical, and UV degradation, including plasticizers, antioxidants, UV stabilizers, lubricants, and pigments (Cousin et al. 2020; Campanale et al. 2020). Since these compounds are not covalently bound to the polymer matrix, they may be released into aquatic systems through leaching processes, potentially affecting aquatic organisms (Campanale et al. 2020; Liu et al. 2020). Studies by Liu et al. (2020) demonstrated that UV stabilizers composed of benzotriazoles caused adverse effects on the freshwater green alga *C. reinhardtii*, suggesting that phytoplankton may also be susceptible to polymer additives.

However, if additive leaching were the only mechanism involved, a progressive increase in toxicity would be expected with increasing MP concentration. In the present study, the highest MP concentration (500 mg L⁻¹) did not result in increased algal inhibition, suggesting that additional processes may have influenced the observed responses. Since algae have a strong affinity to MP surfaces (Bhatt & Chauhan, 2023), higher MP concentrations may have provided a larger available surface area for microorganism colonization and biofilm formation, partially reducing the inhibitory effects observed at lower concentrations. Although the present study did not evaluate physiological parameters such as chlorophyll concentration or gene expression, previous studies have suggested that MPs may impair photosynthesis by affecting these processes (Bhattacharya et al. 2010; Zhang et al. 2017; Prata et al. 2019), due to the additional energetic demand imposed by MPs adsorbed on the cell surface (Lagarde et al. 2016). In addition, particle aggregation at higher concentrations may have altered the interaction between MPs and algal cells, modifying contaminant bioavailability and exposure conditions in the medium. Therefore, at higher MPs concentrations, the larger available surface area may favor biofilm establishment and partially reduce the inhibitory effects observed at lower concentrations.

With regard to comparisons between treatments with MPs alone (5, 50, and 500 mg L⁻¹) and those combined with BaP at the same concentrations, the results of generalized linear models (GLMs) indicated that the toxicity of the treatments with 5 and 50 mg L⁻¹ MPs + BaP did not differ significantly from the respective treatments without PAHs. However, organisms exposed to 500 mg L⁻¹ MPs alone showed reduced toxicity compared to those exposed to 500 mg L⁻¹ + BaP.

Concerning the central hypothesis of this study, the analysis of the combined effects of the two tested contaminants (BaP and MPs) using GLMs indicated the existence of an interaction between the evaluated factors (presence and absence of MPs). Exposure to BaP alone (21 µg L⁻¹) resulted in approximately 57.64% cellular inhibition, differing significantly from the control group (GLM, *p* < 0.05) (Fig. 3). However, when BaP was combined with 50 and 500 mg L⁻¹ MPs, cellular inhibition decreased to approximately 34.85% and 22.78%, respectively, with both treatments differing statistically from BaP alone (GLM, *p* < 0.05). By contrast, the treatment containing 5 mg L⁻¹ MPs + BaP showed inhibition values similar to those observed for BaP alone (58.31%), indicating that under this condition the effects of BaP were not substantially altered. Nevertheless, both treatments remained significantly more toxic than the solvent control (Fig. 3). Although BaP adsorption onto MPs and freely dissolved concentrations were not directly quantified in this study, the results may indicate that, under the experimental conditions evaluated, the interaction between MPs and BaP was insufficient to substantially reduce BaP toxicity in the medium. This finding is of concern because the MP concentration of 5 mg L⁻¹ is environmentally relevant and commonly reported in freshwater environments.

A reduction in PAHs toxicity when combined with high MP concentrations has already been reported in the literature, both for freshwater organisms such as gammarids and cladocerans (Lee et al. 2013; Bartonitz et al. 2020) and for marine organisms such as sea urchins and mysids (de Mello e Souza et al. 2023). These studies indicate that microplastics may reduce the bioavailability of BaP through adsorption processes, thereby temporarily attenuating the immediate effects of the contaminant (Ma et al. 2016). Similar results were described for another PAHs, pyrene. In a study with juvenile *Pomatoschistus microps*, exposure to 200 µg L⁻¹ of pyrene resulted in 100% mortality after 48 h. However, in the presence of MPs, the same level of mortality was only reached after 60 h of exposure, suggesting a delay in the lethal effects of the PAHs (Oliveira et al., 2013). These findings reinforce that MPs can act as vectors for chemical compounds in the environment, not only through the release of their own additives but also by adsorbing hydrophobic contaminants such as BaP present in the medium (Lee et al. 2013).

It is important to note that, although a significant reduction in BaP toxicity was observed at higher MP concentrations in this study, the analyzed effects refer to less sensitive biological responses, such as inhibition of cell growth. However, other studies have demonstrated that even in the absence of effects at higher levels of biological organization (such as amphipod mortality or alterations in sea urchin embryonic development), organisms surviving combined exposures exhibited DNA damage and increased lipid peroxidation (de Mello e Souza et al. 2023).

This study is the first to evaluate the combined effects of BaP and MPs in *Raphidocelis subcapitata*. Unlike previous studies that investigated the isolated toxicity of these contaminants, the present results demonstrate that MPs can modulate BaP toxicity in a concentration-dependent and nonlinear manner, including at environmentally relevant concentrations. These findings contribute to a better understanding of how MPs may alter the bioavailability and ecological risks of PAHs in freshwater primary producers.

## Conclusion

The results of the present study demonstrated that microplastics can alter the toxicological effects of BaP on the chlorophyte microalgae *Raphidocelis subcapitata*. The responses observed indicate that the interaction between MPs and BaP is concentration-dependent, since higher MPs concentrations reduced the cellular inhibition caused by BaP, whereas at the lowest concentration tested (5 mg L⁻¹) the toxic effects remained similar to those observed for BaP alone. These findings suggest that MPs may modify the bioavailability of hydrophobic contaminants in the aquatic environment, probably due to adsorption processes between MPs and BaP.

As primary producers, freshwater microalgae are essential for aquatic ecosystems, acting directly in oxygen production, nutrient cycling, and energy transfer through the food web. Therefore, alterations in algal growth caused by combined exposure to MPs and PAHs may affect ecological processes beyond the individual level. In addition, the results reinforce that MPs cannot be considered inert particles in aquatic systems, since even virgin MPs caused significant effects on algal growth depending on the tested concentration.

Although the present study demonstrated important interactions between MPs and BaP, some limitations should be considered. The adsorption of BaP onto MPs was not directly quantified, and physiological responses such as chlorophyll production, oxidative stress, and gene expression were not evaluated. Thus, additional studies are necessary to better understand the mechanisms involved in the interaction between MPs and PAHs in freshwater organisms. Future research should also investigate chronic effects, trophic transfer, and the responses of other freshwater species exposed to combined contamination scenarios under environmentally realistic conditions.

The present study contributes to improving the understanding of the combined effects of MPs and PAHs in freshwater environments, a topic that remains poorly explored. These findings are relevant considering the increasing occurrence of both contaminants in aquatic ecosystems and their potential transfer through the aquatic food web.

## Supplementary Information

Below is the link to the electronic supplementary material.

Supplementary Material 1

## Data Availability

Data will be available as requested.
