# Supplementary material for: Interactive effects of low-density polyethylene microplastics and benzo[a]pyrene on the growth of the freshwater microalgae Raphidocelis subcapitata
Source: Ecotoxicology. 2026 Sep 1;35(7):161. doi: 10.1007/s10646-026-03143-3 (PMC13534156; doi:10.1007/s10646-026-03143-3)
Supplement: Supplementary file 1 [file 10646_2026_3143_MOESM1_ESM.docx]

Table S1. Composition of the Oligo LC culture medium used for the cultivation of *Raphidocelis subcapitata*, including the calculated final molar concentration of each reagent after dilution in the culture medium.

| **Stock solution** | **Reagent** | **Mass (g)** | **Final volume of stock solution (mL)** | **Volume added per liter of medium (mL)** | **Final concentration in culture medium (M)** |
| --- | --- | --- | --- | --- | --- |
| 1 | Ca(NO_3_)_2_.4H_2_O | 4.0 | 100 | 1 | 1.69 × 10⁻⁴ |
| 2 | KNO_3_ | 10,0 | 100 | 1 | 1.0 × 10⁻^3^ |
| 3 | MgSO_4_.7H_2_O | 3.0 | 100 | 1 | 1.22 × 10⁻⁴ |
| 4 | K_2_HPO_4_ | 4.0 | 100 | 1 | 2.30 × 10⁻⁴ |
| 5 | CuSO_4_.5H_2_O | 0.03 | 1000 | 0.5 | 6.01 × 10⁻⁸ |
|  | (NH_4_)_6_Mo_7_O_24_.4H_2_O | 0.06 |  |  | 2.43 × 10⁻⁸ |
|  | ZnSO_4_.7H_2_O | 0.06 |  |  | 1.04 × 10⁻⁷ |
|  | CoCl_2_.6H_2_O | 0.06 |  |  | 1.26 × 10⁻⁷ |
|  | Mn(NO_3_)_2_.4H_2_O | 0.06 |  |  | 1.20 × 10⁻⁷ |
|  | C_6_H_8_O_7_ | 0.06 |  |  | 1.56 × 10⁻⁷ |
|  | H_3_BO_3_ | 0.06 |  |  | 4.85 × 10⁻⁷ |
| 6 | C_6_H_5_FeO_7_.5H_2_O | 1.625 | 1000 | 0.5 | 1.5 × 10⁻⁶ |
|  | FeCl_3_.6H_2_O | 0.625 |  |  | 1.16 × 10⁻⁶ |
|  | FeSO_4_.7H_2_O | 0.625 |  |  | 1.12 × 10⁻⁶ |
| 7 | NaHCO_3_ | 15.0 | 1000 | 1 | 1.79 × 10⁻⁴ |
